# Intraoperative FCU CMAP Amplitude During Oberlin Nerve Transfer: Association with Reinnervation Timing and Functional Outcomes

**DOI:** 10.3390/jcm15072476

**Published:** 2026-03-24

**Authors:** Diana M. Ortega-Hernández, Isabel Fernández-Conejero, Aroa Casado-Rodríguez, Guillermo J. Tarnawski-Español, Julia Miró-Lladó, Joaquin Casañas-Sintes, Manuel Llusá-Pérez

**Affiliations:** 1 Hand and Microsurgery Unit, Traumaunit, Teknon Medical Center, 08022 Barcelona, Spain; dr.casanas@traumaunit.es; 2 PhD Program in Medicine and Translational Research, Faculty of Medicine and Health Sciences, Universitat de Barcelona, 08036 Barcelona, Spain; 3 Department of Intraoperative Neurophysiology, Bellvitge Universitary Hospital, Universitat de Barcelona, 08907 Barcelona, Spain; isabelfc74@yahoo.es (I.F.-C.);; 4 Human Anatomy and Embryology Unit, Department of Surgery and Medical-Surgical Specialties, Faculty of Medicine (Campus Clínic), Universitat de Barcelona, 08036 Barcelona, Spain; aroa.casado@ub.edu (A.C.-R.); mllusa@ub.edu (M.L.-P.); 5 Orthopaedic Surgery and Traumatology Department, Hospital Fraternidad-Muprespa Habana, 28036 Madrid, Spain; gjtarnawski@gmail.com

**Keywords:** brachial plexus injuries, Oberlin transfer, nerve transfer, ulnar nerve, musculocutaneous nerve, intraoperative neurophysiological monitoring, electromyography, compound muscle action potential

## Abstract

**Background/Objectives**: Selective transfer of an ulnar nerve fascicle to the motor branch of the musculocutaneous nerve (Oberlin technique) is widely used to restore elbow flexion following upper brachial plexus injury. Intraoperative neurophysiological mapping allows quantitative recording of compound muscle action potentials (CMAPs) during donor fascicle selection; however, its prognostic relevance remains unclear. This study evaluated whether intraoperative flexor carpi ulnaris (FCU) CMAP amplitude is associated with time to electromyographic reinnervation of the biceps brachii and with final functional outcomes. **Methods**: A retrospective observational study was conducted including patients who underwent selective nerve transfer to the biceps brachii between 2006 and 2025 at two tertiary referral centers. Donor fascicles were selected using intraoperative neurophysiological mapping with quantitative CMAP recordings from three ulnar-innervated muscles. Primary outcomes were time to electromyographic evidence of reinnervation and final elbow flexion strength assessed using the British Medical Research Council grading system. Associations were analyzed using nonparametric statistical methods. **Results**: Twenty patients met the inclusion criteria. Higher intraoperative FCU CMAP amplitudes were associated with a shorter time to electromyographic reinnervation (Spearman ρ = −0.572, *p* = 0.0106). No association was observed between CMAP amplitude and final elbow flexion strength (Spearman ρ = −0.168, *p* = 0.479), or between time to reinnervation and final functional outcome (Spearman ρ = −0.276, *p* = 0.253). A positive association was found between the injury-to-surgery interval and intraoperative CMAP amplitude (Spearman ρ = 0.681, *p* = 0.000943). **Conclusions**: The intraoperative FCU CMAP amplitude facilitates objective donor fascicle selection and is associated with earlier electromyographic reinnervation. Nevertheless, it was not associated with final elbow flexion strength in this cohort and should be interpreted as a technical adjunct rather than a standalone prognostic indicator. Functional recovery following nerve transfer appears to reflect multifactorial biological and temporal determinants beyond a single intraoperative neurophysiological measurement. These findings should be interpreted cautiously given the limited sample size.

## 1. Introduction

Loss of elbow flexion represents one of the most disabling functional impairments in patients with brachial plexus injuries. The selective nerve transfer described by Oberlin et al. [1] has become a widely adopted technique for restoring biceps brachii function through transfer of an ulnar nerve fascicle to the motor branch of the musculocutaneous nerve. The close anatomical proximity between donor and recipient nerves enables direct neurorrhaphy and shortens the required distance for axonal regeneration, factors consistently associated with favorable clinical outcomes in subsequent series [1,2,3,4,5,6,7,8,9,10]. This approach has also provided the foundation for technical refinements aimed at optimizing postoperative elbow flexion strength [11].

Despite its widespread adoption, appropriate donor fascicle selection within the ulnar nerve remains one of the technically demanding aspects of the procedure. The ulnar nerve is a mixed nerve composed of approximately 52% sensory and 48% motor fibers, as reported by Osman [12]. It exhibits a complex fascicular organization described by Sunderland [13,14] and corroborated in subsequent anatomical studies [8,15,16], characterized by multiple fascicles and interfascicular communications along its course. This intricate architecture, combined with interindividual variability, complicates intraoperative identification of a predominantly motor fascicle directed to the flexor carpi ulnaris (FCU). Accurate selection is intended to minimize the risk of postoperative hand dysfunction [2].

Traditionally, fascicular identification has relied on anatomical landmarks and conventional handheld electrical stimulation, which confirms muscle contraction but does not provide quantitative assessment. The so-called “blocking response” described with this technique may further limit its specificity [3,5,17]. Moreover, the anatomical localization of the fascicle predominantly innervating the FCU has been inconsistently reported in the literature: Teboul [9] described an anterior and medial position, whereas Mackinnon et al. [18] reported a lateral and central location—findings that have also been supported by more recent studies [19].

Intraoperative neurophysiological monitoring has introduced a quantitative dimension to functional nerve assessment. The pioneering work of Kline demonstrated the value of nerve action potential (NAP) recording for early intraoperative evaluation of peripheral nerve injuries [20,21,22]. Within this framework, recording of the compound muscle action potential (CMAP) enables quantification of distal motor responses and assessment of neural continuity. In clinical practice, intraoperative CMAP monitoring has been incorporated to guide fascicular selection during the Oberlin procedure [8,19,23], typically by recording responses from ulnar-innervated muscles such as the flexor carpi ulnaris (FCU), first dorsal interosseous (FDI), and occasionally the abductor digiti minimi (ADM).

Although its technical value in facilitating fascicular localization is generally accepted, limited evidence has examined whether the magnitude of CMAP amplitude correlates with clinically meaningful outcomes, such as time to electromyographic reinnervation of the biceps brachii or ultimate recovery of elbow flexion strength. Existing reports are scarce and heterogeneous, and no clear consensus has been established regarding its prognostic relevance. Demonstration of a true prognostic association would extend its relevance beyond technical guidance, potentially informing intraoperative decision-making. Specifically, CMAP amplitude could influence definitive donor fascicle selection, prompt reconsideration or modification of the reconstructive strategy in the setting of suboptimal responses, and guide the need for adjunctive or alternative nerve transfers.

Moreover, the identification of reliable neurophysiological predictors could refine patient selection criteria and enable early prognostic stratification. Given that functional recovery following nerve transfer is determined by multiple interacting factors—including axonal regeneration distance, duration of target muscle denervation, and subsequent neuromuscular reorganization—clarifying whether intraoperative CMAP amplitude constitutes a meaningful prognostic biomarker, rather than merely a technical adjunct, carries important implications for surgical planning (for example, deciding between single versus double nerve transfer) and individualized patient counseling. However, given the multifactorial biological determinants of recovery, it remains uncertain whether a single intraoperative neurophysiological measurement can independently predict long-term functional outcomes.

The aim of the present study was to evaluate the utility of intraoperative ulnar nerve CMAP recordings as an objective tool for donor fascicle localization and to analyze its relationship with time to electromyographic reinnervation of the biceps brachii and functional outcomes following selective nerve transfer according to the Oberlin technique. The primary objective was to assess the association between intraoperative FCU CMAP amplitude and the time to electromyographic reinnervation, whereas secondary exploratory analyses evaluated its relationship with final functional strength recovery.

## 2. Materials and Methods

### 2.1. Study Design

A retrospective observational study was conducted based on the systematic analysis of a clinical database from two tertiary referral centers in Barcelona, Spain: Hospital Universitari de Bellvitge and Centro Médico Teknon. The study included patients with loss of elbow flexion secondary to upper brachial plexus injury who underwent selective nerve transfer to the biceps brachii using the Oberlin technique between November 2006 and November 2025.

**Figure 1:**
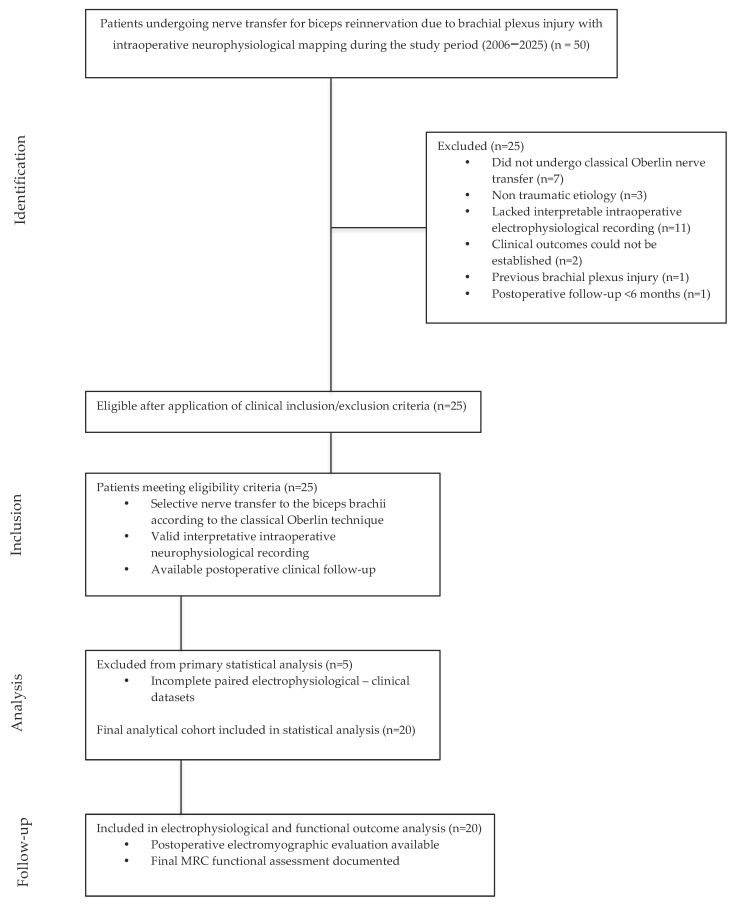
Flow diagram of patient selection and inclusion in the study cohort.

A total of 50 patients were initially identified during the study period. The process of patient identification, eligibility assessment, sequential exclusions, and final cohort derivation is summarized in the STROBE-compliant flow diagram (Figure 1). After application of predefined inclusion and exclusion criteria, 25 patients fulfilled eligibility requirements. Five patients were subsequently excluded due to incomplete paired electrophysiological and clinical datasets, resulting in a final analytic cohort of 20 patients.

In all patients, a single ulnar nerve fascicle with predominant innervation of the FCU muscle was transferred. The donor fascicle was identified intraoperatively with the assistance of neurophysiological monitoring for functional confirmation and precise fascicular localization.

Exclusion criteria were: time from injury to surgery greater than 12 months; postoperative follow-up shorter than 6 months; prior surgical procedures at the level of the surgical approach; pre-existing ulnar nerve lesions; and non-traumatic etiology.

All surgical procedures were performed by the same senior surgeon, and intraoperative neurophysiological assessments were conducted in all cases by the same neurophysiologist, thereby reducing inter-operator variability.

The study protocol was approved by the institutional ethics committees of both participating centers (PR250/18 160518, CMT-2018-UCM01). The design, conduct, and reporting of the study followed the STROBE guidelines for observational studies.

### 2.2. Study Population and Construction of the Analytical Cohort

The initial database comprised 50 consecutive patients who underwent surgical treatment for upper brachial plexus injury at a tertiary referral center. This preliminary cohort reflected real-world clinical practice and included patients with heterogeneous injury patterns, varying surgical techniques, and differing durations of postoperative follow-up.

To establish a homogeneous and methodologically robust analytical cohort aligned with the study objectives, a stepwise data refinement process was undertaken. The process was predefined prior to statistical analysis to minimize selection bias and ensure alignment with the primary study objective. Patients were included only if they met all of the following criteria:

- Underwent selective nerve transfer to the biceps brachii according to the classical Oberlin technique.
- Have an intraoperative electrophysiological recording of the donor fascicle CMAP amplitude from the FCU muscle.
- Have at least one postoperative recording of the CMAP of the ulnar nerve in any target muscle.
- Have a documented postoperative functional assessment.

Patients were excluded if they underwent end-to-side or supercharge nerve transfers, had a non-traumatic etiology of brachial plexus injury, lacked interpretable intraoperative electrophysiological recordings, had insufficient clinical follow-up, or if a reliable correspondence between electrophysiological data and clinical outcomes could not be established.

After application of these criteria, 25 patients fulfilled eligibility requirements. Five patients were subsequently excluded from the primary analysis due to incomplete electrophysiological or clinical data that precluded reliable paired statistical evaluation.

Following this refinement process, the final analytic sample for the primary analysis consisted of 20 patients with complete datasets.

The effective sample size varied across secondary analyses depending on data completeness, and the exact number of cases included in each statistical test is explicitly reported in the corresponding tables to ensure analytical transparency.

### 2.3. Intraoperative Neurophysiological Recording

The standard surgical approach for nerve transfer to the biceps muscle was performed as previously described [7]. Following incision along the bicipital groove, the ulnar and musculocutaneous nerves were identified. In cases involving limb ischemia, the tourniquet was deflated and at least half of the ischemia time was allowed to elapse prior to recording to ensure adequate reperfusion and restoration of neural conductivity.

NAP was recorded from the musculocutaneous nerve. Distal stimulation of the ulnar nerve was then performed on a fascicle-by-fascicle basis, with CMAPs recorded from three ulnar-innervated muscles: FDI, ADM and FCU. The amplitude of the FCU CMAP (baseline-to-negative peak, expressed in μV) was quantitatively measured and recorded for subsequent statistical analysis. The donor fascicle was selected based on the highest baseline-to-negative peak amplitude recorded in the FCU, with minimal or absent contribution to the intrinsic hand muscles (Video S1).

Intraoperative monitoring was conducted using a 32-channel ISIS IOM system (INOMED^®^, Emmendingen, Germany). Electrical stimulation was delivered via bipolar or tripolar probes (interelectrode spacing 3–5 mm for small nerves or fascicles and 5–7 mm for larger nerves), using square-wave pulses of 0.05–0.2 ms duration, cathodal polarity, frequencies of 1–3 Hz, and stimulation intensities ranging from 0.1 to 5 mA. Recordings were obtained with bipolar subdermal needle electrodes (12 mm/27G) placed at the motor point, with an interelectrode distance of 30 mm for FCU and 20 mm for FDI and ADM. A sweep duration of 30 ms and sensitivity of 50 μV–5 mV/div were used, with high-pass filters set at 5–10 Hz and low-pass filters at 2000–3000 Hz. All recordings were obtained under standardized total intravenous anesthesia (TIVA) without neuromuscular blocking agents to avoid interference with CMAP recordings.

Following segmental and limited intraneural neurolysis, repeated CMAP verification and NAP measurements were performed. The selected fascicle was marked, mobilized to the required length, and transferred to the motor branch of the biceps muscle. Intraoperative CMAP values were documented prospectively in the operative report or, when available, in a dedicated intraoperative neurophysiology report. During the retrospective analysis, these values were extracted from the records and, when necessary, verified directly from the original neurophysiological recordings by the neurophysiologist who performed the intraoperative monitoring.

### 2.4. Postoperative Electrophysiological Assessment

Available electromyographic studies performed during routine postoperative follow-up were reviewed. The electrophysiological variables analyzed included the following:

- Presence of electromyographic signs of reinnervation.
- Time to electromyographic evidence of reinnervation of the biceps brachii.
- CMAP amplitude of the ulnar nerve recorded from the FCU, FDI, and ADM, when available.
- Time to the most recent electromyographic evaluation.

Electromyographic evidence of reinnervation was defined as the first postoperative EMG demonstrating nascent or polyphasic motor unit potentials in the biceps brachii consistent with axonal reinnervation, with or without voluntary recruitment.

Time to reinnervation was defined as the interval between the date of surgery and the date of the first postoperative EMG demonstrating these findings in the biceps brachii. The interval was expressed in both days and months to facilitate temporal comparisons and longitudinal analyses. Both dates were recorded in the clinical records using the day–month–year format, allowing the interval to be calculated precisely in days.

All available postoperative EMG reports were systematically reviewed, and when multiple studies were available, the earliest examination fulfilling the reinnervation criteria was considered for analysis.

### 2.5. Functional Assessment

Final functional outcome was evaluated using the British Medical Research Council (MRC) grading system, a widely accepted ordinal scale for the assessment of muscle strength in peripheral nerve surgery. The MRC scale ranges from 0 (no contraction) to 5 (normal strength), and assessments were obtained during routine postoperative clinical follow-up.

For selected analyses, a clinically meaningful threshold for functional recovery was defined as an MRC grade ≥ 3, corresponding to the ability to achieve active movement against gravity. Given the ordinal nature and limited granularity of the MRC scale, results were interpreted with caution, particularly in subgroup comparisons involving small sample sizes.

### 2.6. Additional Clinical Variables

The following variables were analyzed as potential modulators of outcome:

- Age at the time of surgery.
- Time interval from injury to surgical intervention.
- Injury pattern, categorized as upper trunk involvement (C5–C6 or C5–C6–C7) versus other lesion patterns.
- Type of donor fascicle, classified as pure or mixed based on the distribution of recorded muscle responses.
- Performance of additional reconstructive procedures during the same surgical session.

These variables were explored in secondary, hypothesis-generating analyses to assess their potential influence on electrophysiological and functional outcomes.

### 2.7. Statistical Analysis

Continuous variables were summarized using minimum, maximum, and median values. For non-normally distributed variables, the interquartile range (IQR) was additionally reported to provide a robust measure of dispersion.

Given the limited sample size and the non-normal distribution of several variables, nonparametric statistical methods were used throughout the analysis. Associations between continuous variables were assessed using Spearman’s rank correlation coefficient, with reporting of the correlation coefficient (ρ) and corresponding *p* value. For each analysis, the exact sample size (n) was specified.

The association between intraoperative FCU CMAP amplitude and time to electromyographic reinnervation was defined a priori as the primary analysis. Comparisons between subgroups were performed using the Mann–Whitney U test, with reporting of the U statistic and exact *p* value. All additional correlations and subgroup analyses were considered exploratory and hypothesis-generating.

Multivariable analyses were not conducted due to the limited sample size in order to minimize the risk of statistical overfitting. To explore potential confounding by the injury-to-surgery interval, a partial Spearman correlation was performed to assess the relationship between FCU CMAP amplitude and time to reinnervation while controlling for this variable.

A *p* value < 0.05 was considered statistically significant. Given the limited sample size and the number of exploratory analyses performed, findings should be interpreted with caution, and no formal correction for multiple comparisons was applied. The study was not powered to detect small to moderate effect sizes for secondary analyses.

Statistical analyses were performed using Jamovi software (version 2.7.17), current at the time of analysis.

## 3. Results

The final analytical cohort comprised 20 patients who underwent selective nerve transfer to the biceps brachii, with valid intraoperative neurophysiological recordings and available postoperative clinical and electrophysiological follow-up. Although 25 patients fulfilled eligibility criteria, four were excluded from the primary analysis due to incomplete paired datasets. Demographic characteristics are summarized in Table S1. Six patients (30%) exhibited an unfavorable functional outcome (MRC < 3), whereas 14 (70%) achieved satisfactory functional recovery (MRC ≥ 3).

The intraoperative CMAP amplitude recorded from the flexor carpi ulnaris (FCU) demonstrated a non-normal distribution and is therefore reported as median and interquartile range. The median FCU CMAP amplitude was 600 μV (IQR 250–1800; range 100–7000 μV). The anatomical distribution of fascicles demonstrating predominant CMAP responses in the FCU is detailed in Table S2. The intraoperative NAP amplitude had a median of 100 μV (IQR 80–200; range 60–400 μV).

The mean time to electromyographic evidence of reinnervation was 162.9 ± 46.9 days (median 170; range 71–265), corresponding to 4.79 ± 1.47 months (median 5; range 2–8). The interval from injury to surgery was 7.07 ± 2.83 months (median 6.54; range 4.60–9.92).

Correlation analyses are summarized in Table 1. No significant association was observed between intraoperative FCU CMAP amplitude and final functional outcome (ρ = −0.168; *p* = 0.479). Similarly, CMAP amplitude did not correlate with age (ρ = −0.280; *p* = 0.231).

In contrast, higher intraoperative FCU CMAP amplitudes were significantly associated with shorter time to reinnervation, both when expressed in days (ρ = −0.572; *p* = 0.0106) and in months (ρ = −0.511; *p* = 0.0254) (Table 1). Age was not associated with time to reinnervation (ρ = 0.361; *p* = 0.129), and no correlation was identified between the injury-to-surgery interval and reinnervation time (ρ = −0.276; *p* = 0.253).

A significant positive association was observed between the interval from injury to surgery and the intraoperative CMAP amplitude recorded from the flexor carpi ulnaris (FCU) (ρ = 0.681; *p* = 0.000943). However, the injury-to-surgery interval was not associated with final functional outcome (ρ = −0.125; *p* = 0.600). Time to reinnervation did not correlate with functional outcome (ρ = −0.199; *p* = 0.413). Likewise, no significant association was identified between intraoperative NAP amplitude and FCU CMAP amplitude (ρ = 0.155; *p* = 0.581) (Table 1).

To explore potential confounding, a partial Spearman correlation analysis was performed to assess the relationship between intraoperative FCU CMAP amplitude and time to reinnervation (days) while controlling for the injury-to-surgery interval. The association remained statistically significant after adjustment (partial ρ = −0.474; *p* = 0.040; n = 19).

Follow-up duration demonstrated a significant positive correlation with final MRC grade (ρ = 0.714; *p* = 0.0004; n = 20; 95% CI, 0.466–0.881), indicating that longer follow-up was associated with improved functional recovery.

Subgroup analyses are presented in Table 2. Patients operated on more than six months after injury exhibited significantly higher FCU CMAP amplitudes compared with those treated within ≤6 months (U = 21.0; z = −2.088; *p* = 0.0387). No significant differences were observed according to injury pattern (*p* = 0.750), donor fascicle type (*p* = 0.3749), combined versus isolated procedures (*p* = 0.3847), or functional outcome category (*p* = 0.7791). All subgroup comparisons should be interpreted as exploratory analyses.

Overall, intraoperative FCU CMAP amplitude was associated with longer injury-to-surgery intervals and earlier reinnervation, but not with final functional recovery.

No weakness in hand function was reported by any of the patients.

## 4. Discussion

The present study was not designed to demonstrate the superiority of intraoperative neurophysiological monitoring but rather to critically evaluate whether intraoperative FCU CMAP amplitude carries prognostic value in selective nerve transfer using the Oberlin technique and to clarify its practical clinical relevance. Specifically, we examined its association with time to electromyographic reinnervation, final elbow flexion strength, and its utility for donor fascicle localization. The association between FCU CMAP amplitude and time to reinnervation was predefined as the primary analysis, whereas all additional comparisons were considered exploratory.

Intraoperative peripheral nerve monitoring has long been incorporated into surgical practice for nerve identification, lesion characterization, functional continuity assessment, detection of avulsion injuries, and prevention of iatrogenic injury [24,25,26]. However, its role as a quantitative prognostic indicator in selective nerve transfer remains insufficiently defined. Our findings suggest that intraoperative FCU CMAP amplitude reflects the functional integrity of the donor nerve at the time of surgery; however, it does not reliably predict postoperative functional recovery.

Reinnervation following the Oberlin transfer has consistently been reported at approximately 3–4 months postoperatively [2,3,27], and our results align with these observations. Although higher intraoperative FCU CMAP amplitudes were significantly associated with earlier electromyographic evidence of reinnervation (Spearman ρ = −0.572, *p* = 0.0106), time to reinnervation itself did not correlate with final elbow flexion strength. Importantly, although this association persisted after controlling for the injury-to-surgery interval, the confidence interval of the partial correlation was wide and included values near zero, reflecting statistical uncertainty inherent to the limited sample size. It should be emphasized that this association reflects a statistical relationship rather than a causal inference. Given the exploratory design and absence of multivariable adjustment, these findings should be interpreted cautiously and considered hypothesis-generating.

A particularly relevant finding was the strong positive correlation between follow-up duration and final MRC grade (ρ = 0.714). This indicates that patients with longer follow-up tended to achieve higher functional grades, suggesting that some individuals with shorter follow-up may not yet have reached maximal recovery at the time of evaluation. Consequently, the absence of an association between intraoperative CMAP amplitude and final strength outcome may partly reflect temporal heterogeneity in follow-up rather than a definitive absence of biological relationship.

These observations support the multifactorial nature of muscle recovery after nerve transfer. Functional outcome depends not only on the onset of reinnervation, but also on duration of denervation and motor endplate preservation [28,29,30], biological condition of the recipient muscle [31,32], donor nerve axonal load and regenerative capacity [23,33,34], regeneration distance [1,35], patient age [31,36,37,38,39,40], and systemic factors [31,40]. Experimental and clinical evidence indicates that motor endplate degeneration becomes progressively irreversible after approximately six months of denervation, with marked structural deterioration between 12 and 18 months [5]. In this context, a single intraoperative neurophysiological measurement is unlikely to fully reflect the complex biological processes that ultimately govern functional recovery.

Although early surgical intervention is generally associated with improved outcomes—as described by Chuang [29] and partially supported by Teboul et al. [9] and others [3,5,41]—the absence of association between surgical delay and functional outcome in our cohort should be interpreted with caution. The relatively homogeneous timing of surgery and limited sample size may have reduced the ability to detect subtle timing effects. Therefore, our findings do not contradict prior literature but suggest that surgical timing alone may not fully explain outcome variability within this clinical context.

Interestingly, time from injury to surgery showed a positive association with intraoperative FCU CMAP amplitude. This relationship was explored because the injury-to-surgery interval could potentially act as a confounding factor in the association between donor nerve physiological status and time to reinnervation. The observed correlation may reflect partial preservation or spontaneous recovery of donor motor units in selected cases. However, surgical timing was not associated with final outcome according to the MRC scale, reinforcing that donor nerve physiological status alone does not determine definitive functional restoration. Recovery likely depends on the complex interplay between donor nerve biology, recipient muscle viability, and central neuroplastic adaptation, rather than on the absolute donor CMAP amplitude at the time of transfer.

Our anatomical mapping findings further clarify the technical value of intraoperative CMAP-guided fascicular selection. Classical descriptions of ulnar nerve fascicular organization remain debated. Sunderland [14] and Llusa et al. [16] demonstrated extensive interfascicular interconnections, particularly in proximal segments, challenging the concept of stable motor–sensory segregation. Although Oberlin later suggested that intraneural mapping may allow identification of motor-dominant fascicles [42], our results confirm substantial anatomical variability. No consistently isolated fascicle exclusively directed to the FCU was identified, and mixed innervation patterns were common. These findings reinforce the practical importance of quantitative intraoperative mapping while not allowing definitive conclusions regarding fascicular organization patterns.

In this context, no patient in our cohort developed a postoperative motor deficit involving the intrinsic hand muscles. Although definitive conclusions cannot be drawn from a limited sample, this observation suggests that CMAP-guided donor fascicle selection may contribute to preserving intrinsic motor function when fascicles with a relatively greater extrinsic motor fiber contribution are preferentially chosen. Given the mixed composition of the ulnar nerve and the well-documented presence of interfascicular cross-connections, strict functional segregation of motor subcomponents cannot be assumed. The transient dysesthesias observed in a small number of patients may therefore reflect the inherent mixed fascicular architecture of the ulnar nerve rather than inaccurate motor fascicle identification. Taken together, these findings are consistent with the functional safety of intraoperative neurophysiological mapping within selective nerve transfer procedures, while acknowledging the anatomical complexity of ulnar nerve organization.

In addition, intraoperative FCU CMAP amplitude was not associated with the anatomical level of injury, suggesting that selective nerve transfer may remain technically feasible even in more extensive lesion patterns, provided that donor nerve functional integrity is preserved. This observation may expand the potential applicability of the technique and supports individualized intraoperative neurophysiological assessment rather than rigid exclusion criteria based solely on injury pattern.

With regard to strength recovery, previous studies have suggested that the electrophysiological status of the donor nerve may influence postoperative strength [31,43], although heterogeneous methodologies limit direct comparison. In our series, intraoperative FCU CMAP amplitude was not associated with final elbow flexion strength. It should be acknowledged that functional outcome was assessed using the ordinal MRC scale, which has limited granularity and may lack sensitivity to detect subtle differences in strength recovery. Standardized quantitative strength measurements (e.g., dynamometry) were not systematically available for all included patients. This methodological limitation should be considered when interpreting the absence of a detectable association between intraoperative CMAP amplitude and final elbow flexion strength. The absence of a detectable association should be interpreted within the limitations of sample size and statistical power and does not necessarily imply the absence of a biological effect. Intraoperative CMAP amplitude quantifies recruitable motor units at a single time point but does not capture axonal sprouting dynamics, synaptic integration efficiency, cortical reorganization, or the biological status of the recipient muscle. Consequently, its role as a standalone prognostic biomarker appears conceptually limited.

From a clinical standpoint, these findings indicate that intraoperative FCU CMAP amplitude should be interpreted within the broader surgical and biological context. Although it provides objective information regarding donor nerve excitability and facilitates safe fascicular selection, it appears insufficient, when considered in isolation, to predict final motor recovery or establish prognosis. Quantitative intraoperative neurophysiological parameters may therefore complement—but should not replace—comprehensive anatomical assessment and informed surgical judgment during selective nerve transfer procedures.

Certain limitations should be considered when interpreting these findings, including the retrospective design and limited sample size. Multivariable modeling was not performed due to concerns of overfitting, and potential confounders were therefore not simultaneously adjusted for. Effective sample size varied across analyses due to data completeness, and the reduction from the initially assessed population to the final analytic cohort may introduce selection bias, as illustrated in the STROBE flow diagram. Follow-up duration varied substantially across patients and may not have captured complete motor unit stabilization in all cases. Furthermore, the cohort consisted exclusively of male patients, reflecting the epidemiological distribution of traumatic upper brachial plexus injuries but potentially limiting generalizability to female populations. Accordingly, the findings should be interpreted within an exploratory clinical framework. Nevertheless, the study reflects standardized surgical and neurophysiological protocols applied in high-volume tertiary centers, providing clinically relevant real-world data.

Prospective studies integrating quantitative intraoperative metrics with standardized objective strength measurements and multivariable analysis may further clarify the relationship between donor nerve physiology and long-term functional recovery. The methodological approach described here may also be applicable to other selective nerve transfer procedures, potentially facilitating more standardized integration of intraoperative neurophysiological data into surgical decision-making.

## 5. Conclusions

Intraoperative FCU CMAP amplitude provides objective information regarding donor nerve functional integrity and supports precise fascicular localization during selective nerve transfer according to the Oberlin technique. In this exploratory cohort, higher amplitudes were associated with earlier electromyographic reinnervation but were not independently associated with final elbow flexion strength.

These findings suggest that intraoperative CMAP amplitude should be regarded primarily as a technical and adjunctive intraoperative parameter rather than as a definitive prognostic marker. Its prognostic interpretation should therefore account for follow-up duration, biological determinants of recovery, and the inherent uncertainty observed in small exploratory cohorts. In clinical practice, intraoperative CMAP measurements may therefore support safe donor fascicle selection while complementing, rather than replacing, comprehensive anatomical assessment and surgical judgment.

## Figures and Tables

**Table 1:** Spearman Correlations Between FCU CMAP, Temporal Variables, and Clinical Outcomes.

Comparison	ρ	95% CI (Bootstrap)	n	*p*-Value
FCU CMAP vs. surgical delay	0.681	0.456 to 0.843	20	0.0009
FCU CMAP vs. reinnervation (days)	−0.572	−0.822 to −0.202	19	0.0106
FCU CMAP vs. reinnervation (days) *	−0.474	−0.846 to 0.061	19	0.0405
FCU CMAP vs. MRC	−0.168	−0.588 to 0.289	20	0.4794
FCU CMAP vs. age	−0.280	−0.593 to 0.064	20	0.2312
Age vs. reinnervation (days)	0.361	−0.163 to 0.763	19	0.1292
Surgical delay vs. MRC	−0.125	−0.494 to 0.235	20	0.6001
Surgical delay vs. reinnervation (days)	−0.276	−0.645 to 0.113	19	0.2529
Reinnervation (days) vs. MRC	−0.199	−0.681 to 0.300	19	0.4130
NAP vs. FCU CMAP	0.155	−0.444 to 0.688	15	0.5813
Follow-up duration vs. MRC	0.714	0.466 to 0.881	20	0.0004

* Partial correlation controlling for injury-to-surgery interval. Note: 95% confidence intervals estimated using bootstrap resampling (5000 iterations). Abbreviations: CMAP, compound muscle action potential; FCU, flexor carpi ulnaris; NAP, nerve action potential; MRC, British Medical Research Council muscle strength grading system.

**Table 2:** Subgroup Comparisons of Intraoperative FCU CMAP Amplitude (Mann–Whitney U Test).

Comparison	n_1_	n_2_	U	z	*p*-Value
Surgical delay ≤ 6 months vs. >6 months	9	11	21.0	−2.088	0.0387
Upper trunk vs. other patterns	12	8	28.0	−0.379	0.750
Pure vs. mixed fascicle	8	12	49.0	0.953	0.3749
Isolated vs. combined procedure	13	7	22.0	−0.947	0.3847
Functional outcome (MRC ≥ 3 vs. <3)	14	6	38.0	−0.331	0.7791

Note: All subgroup analyses are exploratory. Abbreviations: MRC, British Medical Research Council muscle strength grading system.

## Data Availability

The datasets generated and/or analyzed during the current study are not publicly available due to patient privacy and ethical restrictions but are available from the corresponding author upon reasonable request and subject to Institutional Review Board approval.

## Supplementary Materials

The following supporting information can be downloaded at https://www.mdpi.com/article/10.3390/jcm15072476/s1. Table S1. Baseline demographic and clinical characteristics of the analytical cohort and stratified by injury level (n = 20); Table S2. Intraoperative Donor Fascicle Characteristics; Video S1. Donor fascicle localization and selection during intraoperative neurophysiological mapping prior to intraneural neurolysis in the Oberlin nerve transfer procedure.

## Abbreviations

The following abbreviations are used in this manuscript:

CMAP	Compound muscle action potential
NAP	Nerve action potential
FCU	Flexor carpi ulnaris
FDI	First dorsal interosseous
ADM	Abductor digiti minimi
MRC	British Medical Research Council muscle strength grading system
