# Supplementary material for: Intraoperative FCU CMAP Amplitude During Oberlin Nerve Transfer: Association with Reinnervation Timing and Functional Outcomes
Source: J Clin Med. 2026 Mar 24;15(7):2476. doi: 10.3390/jcm15072476 (PMC13073934; doi:10.3390/jcm15072476)
Supplement: Supplementary file 1 [file jcm-15-02476-s001.zip › jcm-4188078-Supplementary materials (tables).pdf]

Supplementary Table S1. Baseline demographic and clinical characteristics of the analytical cohort and stratified by injury level (n = 20)

**Variable**	**Value**
Age at first visit (years), mean ± SD	36.95 ± 14.48
Age at first visit (years), median (IQR)	37.5 (27.0–46.0)
Sex, n (%)	
— Male	20 (100)
— Female	0 (0)
Affected side, n (%)	
— Left	14 (70)
— Right	6 (30)
Time from injury to surgery (months), mean ± SD	7.07 ± 2.83
Time from injury to surgery (months), median (IQR)	6.54 (4.60–9.92)
Follow-up duration (months), mean ± SD	27.51 ± 22.76
Follow-up duration (months), median (IQR)	19.61 (13.21–30.01)
Concomitant nerve transfers performed, n (%)	15 (75)
Postoperative dysesthesias, n (%)	2 (10)

Abbreviations: SD, standard deviation; IQR, interquartile range.

Baseline characteristics stratified by level of brachial plexus injury (n = 20)

Injury Level	n	Age (years), mean ± SD	Time to Surgery (months), mean ± SD
**C5–C6**	6	31.4 ± 12.4	8.3 ± 1.6
**C5–C6–C7**	12	36.9 ± 12.1	5.9 ± 2.4
**C5–C6–C7–C8**	2	32.3 ± 10.2	7.5 ± 3.8

Abbreviations: SD, standard deviation.

Notes:

- Values are presented as mean ± SD for clinical comparability across injury patterns.
- Small subgroup sizes—particularly in the C5–C6–C7–C8 group—limit distributional assumptions and preclude meaningful inferential comparisons.

Supplementary Table S2. Intraoperative Donor Fascicle Characteristics

(A) Fascicle Composition Based on Intraoperative Mapping (n = 18 evaluable cases)

Fascicle Type	n	%
**Pure FCU**	7	38.9
**FCU + FDI**	2	11.1
**FCU + FDI + ADM**	9	50.0

(B) Anatomical Localization of the Donor Fascicle (n = 14 precisely mapped cases)

Anatomical Location	n	%
**Anterolateral**	4	28.6
**Anteromedial**	2	14.3
**Posterolateral**	4	28.6
**Posteromedial**	3	21.4
**Medial**	1	7.1

Percentages are calculated according to evaluable cases in each subgroup.

Abbreviations: FCU, flexor carpi ulnaris; FDI, first dorsal interosseous; ADM, Abductor digiti minimi.

Note: Some cases lacked complete intraoperative documentation or detailed operative records describing fascicular composition or precise anatomical localization. Therefore, fascicle composition could be evaluated in 18 cases and precise anatomical localization in 14 cases.

Video S1. Donor fascicle localization and selection during intraoperative neurophysiological mapping prior to intraneural neurolysis in the Oberlin nerve transfer procedure

Video S1 is provided as supplementary material associated with this article.
